# The Efficacy of Psychological Therapies in Reducing Weight and Binge Eating in People with Bulimia Nervosa and Binge Eating Disorder Who Are Overweight or Obese—A Critical Synthesis and Meta-Analyses

**DOI:** 10.3390/nu9030299

**Published:** 2017-03-17

**Authors:** Marly Amorim Palavras, Phillipa Hay, Celso Alves dos Santos Filho, Angélica Claudino

**Affiliations:** 1 Eating Disorders Program (PROATA), Department of Psychiatry, Federal University of São Paulo (UNIFESP), São Paulo 04038-000, Brazil; marlypalavras@gmail.com (M.A.P.); celsoalvesfilho@uol.com.br (C.A.d.S.F.); angelica.claudino@uol.com.br (A.C.); 2 CAPES Foundation, Ministry of Education of Brazil, Brasilia 70047-900, Brazil; 3 School of Medicine, Western Sydney University, Sydney 2751, Australia; 4 Centre for Health Research, School of Medicine, Western Sydney University, Sydney 2751, Australia

**Keywords:** obesity, binge eating, psychotherapy, weight management

## Abstract

Recurrent binge eating episodes, the core feature of Bulimia Nervosa (BN) and Binge Eating Disorder (BED), are frequently comorbid with obesity. Psychological interventions, notably Cognitive Behavioural Therapy (CBT), are effective for binge eating reduction in BED or BN but less so for weight loss. Behavioural Weight Loss Therapy (BWLT) shows effectiveness for binge eating reduction and weight loss but the latter appears poorly sustained over time. Our aim was to review evidence for efficacy of psychological therapies for BN/BED associated with overweight or obesity in reducing binge frequency and weight. A systematic search for randomized controlled trials with adult samples who had BN or BED was conducted considering articles in English, French, Spanish and Portuguese with no restrictions for the timeline publication ending in March 2016. A quality appraisal of the trials and meta-analyses comparing BWLT to CBT were done. This review identified 2248 articles for screening and 19 published articles were selected. No trials of BN were identified. This review found CBT was favoured compared to BWLT with regard to short-term binge eating reduction. However, insufficient evidence was found for superiority for BWLT efficacy compared to CBT considering binge eating remission, reduction of binge eating frequency and weight loss. More research is needed to test the efficacy of psychological treatments for BED or BN with co-morbid overweight or obesity, including trials evaluating binge eating remission and weight loss in the long-term.

## 1. Introduction

Eating disorders (EDs) are severe conditions that occur across age groups and in both sexes [1]. Worldwide, they occur most frequently in young women, with prevalence rates that can be up to 10% in community populations [2,3]. The most common are Bulimia Nervosa (BN) and Binge Eating Disorder (BED) [3,4]. Recurrent binge eating episodes are the core feature of both conditions and these are characterized by the consumption of an unusually large amount of food associated with the sense of loss of control over eating during the episode [5]. BN and recurrent binge eating are both increasing and are associated with considerable impairment, both psychological and physical, including overweight or obesity [2,6,7,8]. The recently published Noncommunicable Diseases NCD Risk Factor Collaboration that used 1698 population-based data sources encompassing 186 countries reported large increases in global age-standardised mean Body Mass Index (kg/m^2^, BMI) from 1975 to 2014 [9]. As described by the researchers, in this period, men’s mean BMI changed from 21.7 kg/m^2^ to 24.4 kg/m^2^, and women’s mean BMI increased from 22.1 kg/m^2^ to 24.4 kg/m^2^. If this trend persists, the authors estimate that, in 2025, the global obesity prevalence will be 18% in men and above 21% in women. The relationship between binge eating and obesity is complex and likely bidirectional [10]. An Australian community study that examined the increase in prevalence rates of ED’ behaviours and obesity over the 10-year period from 1995 to 2005 reported that rates of obesity associated with ED’ behaviours increased from 8.5% to 20% [7]. The findings of increasing rates of these two conditions together support the need for the development of effective approaches to prevent and to treat them, especially when they co-occur.

In a 2012 study, Bulik et al. [11] reported that during the course of a trial comparing group Cognitive Behavioural Therapy (CBT) versus online CBT for patients with BN, there was an increase in weight, with individuals reaching the overweight or obesity ranges. The authors argued that in face of the rise of obesity worldwide the appearance of overweight or obesity in individuals with BN should not be unexpected. Indeed, the National Comorbidity Survey Replication reported a point prevalence of 20.9% for BMI ≥ 30 kg/m^2^ and 10.3% for BMI ≥ 40 kg/m^2^ in individuals with a lifetime diagnosis of BN [12].

The treatment of obesity is challenging. The Australian Guidelines for the management of obesity consider the most effective treatment (based on a weight loss above 10% of baseline weight that is maintained over five years) is achieved by bariatric surgery; moderate effectiveness is found with pharmacotherapy plus lifestyle change (diet, physical activity or psychological therapy) and lifestyle change alone has the lowest effectiveness [13]. As for EDs, a systematic review examined available psychological therapies and concluded that the best evidence for the treatment of BN was a therapist-led CBT [14]. For BED, CBT in full or as guided self-help intervention had the greatest support, but for patients with BED associated with obesity, behaviour weight loss therapy (BWLT) appeared to be an alternative to CBT, at least in the short-term [14]. A further systematic review reported that even very low calorie diets may be beneficial for individuals with BED [15]. Another recent review found CBT and Interpersonal Therapy (IPT) to be the most effective treatments for binge eating reduction over the short and long term for patients with BED [16]. However, these last two approaches do not target weight loss. On the other hand, BWLT has shown effectiveness for binge eating reduction associated with weight loss in the short term, but this was poorly sustained over time [17]. 

Hence, the aim of this systematic review was to investigate the efficacy of psychological therapies for the treatment of BN and BED associated with overweight or obesity, with a special focus on the effects of BWLT as this is the only therapy currently developed to reduce both binge eating and weight. We planned meta-analyses of studies that compared BWLT versus no treatment or wait-list, and BWLT versus another psychological therapy. The primary outcomes of interest in the meta-analyses were binge eating frequency and weight/BMI. As weight loss in obesity trials is now routinely reported to one year, this was a second specific outcome [18] and a third was treatment completion as attrition is a source of bias and completion may be a proxy for acceptability.

## 2. Materials and Methods

### 2.1. Literature Search Strategies

The Preferred Reporting Items for Systematic Review and Meta-Analyses (PRISMA) guideline was used for the conduct of this systematic review [19]. A search of published academic journal articles was conducted in indexed journals of seven electronic databases: MEDLINE, PsycINFO, WEB OF SCIENCE, SCOPUS, COCHRANE, EMBASE E LILACS from 18 January 2016 to 8 March 2016 using the following terms: (bulimia nervosa OR binge eating disorder OR eating disorders OR bulimia OR binge eating OR binging) NOT (anorexia nervosa OR binge drinking) AND (overweight OR obesity) AND (psychotherapy OR therapeutics OR cognitive therapy OR psychological therapy OR psychological treatment) NOT (drug therapy OR pharmacological treatment). 

Additional searches were made through the reference list of papers selected. Unpublished papers were searched through the ProQuest Dissertations and Theses in Western Sydney University library in 21 October 2016.

### 2.2. Selection Criteria

The inclusion criteria for studies in this review were randomized controlled trials that investigated the efficacy of behavioural weight loss and other psychological therapies for the treatment of individuals with BED or BN associated with overweight or obesity. Studies that evaluated psychological interventions such as CBT, Cognitive Behavioural Therapy-Enhanced (CBT-E), IPT, BWLT, Behaviour Therapy, short-term focal psychotherapy, Dialectical Behaviour Therapy (DBT), self-help therapy, analytic psychotherapy, meditation, mindfulness, or any other psychological therapy were included. Trials that compared two or more forms of psychological therapy or a psychological therapy compared to a wait-list/delayed treatment or to placebo were included. Only articles in English, French, Spanish and Portuguese were considered with no restrictions for the timeline publication. Three authors (MAP, CASF, and AC) are fluent in Portuguese, written Spanish and French, and a fourth is a native English speaker (PH).

Trials included in this review involved: (i) adults (≥18 years old) of both genders; (ii) individuals diagnosed with BN, or BED, or Eating Disorder Not Otherwise Specified (EDNOS)-BN/BED type [20], or Other Specified Feeding or Eating Disorder (OSFED)-BN/BED according to a valid diagnostic scheme such as the Diagnostic and Statistical Manual of Mental Disorders, Fourth Edition, Text Revised (DSM-IV-TR) [20], or DSM-5 [5], or the International Statistical Classification of Diseases and Related Health Problems 10th Revision (ICD-10) [21], or the proposed ICD-11 [22], based either on a validated instrument (e.g., Eating Disorder Examination) [23], or specialist clinician judgment; (iii) studies where at least 90% of the participants had a BMI ≥ 27 kg/m^2^; and (iv) patients treated in primary, secondary or tertiary outpatient care. Exclusion criteria included studies developed in inpatient or partial hospital facilities where it is not possible to determine specifically the effect of the psychological approach in a complex multi-treatment environment and studies examining secondary questions such as predictors of outcomes (termed “secondary studies”). Literature reviews, book chapters, conference annals, theoretical articles, and studies of pharmacological treatment, bariatric surgery, exercise alone or another non-psychological therapy were also excluded.

Two reviewers (MAP and CASF) independently examined the titles and abstracts of the citations that emerged from the researches and selected articles to be fully reviewed by both for the definition of those that fulfilled the purposes of this review. Two further senior authors’ opinions (AMC and PH) were requested when consensus was not achieved by the two reviewers. All the authors are clinicians and researchers in the field of EDs.

Data extracted from papers were: year of publication, demographic features of participants, participant diagnoses and method of ascertainment, sample size and interventions’ time points of assessment. Data were extracted by MAP and checked for accuracy by the other authors.

### 2.3. Quality Assessment

Data extracted for appraisal of trial quality were the method of diagnostic ascertainment and main outcome of binge frequency, sample size and a priori power analysis, risk of bias, blinding, and length of follow-up (≥12 months). A consensus on trial quality was made between two authors (MAP and PH). The overall risk of bias across studies was defined as: (i) high when the information of the studies at risk of bias was sufficient to affect the interpretation of the results; (ii) low when the available information was sufficient not to affect the interpretation of the results; and (iii) unclear when the information was not clearly specified or even reported. Attrition >20% was considered moderate and >50% was considered high [24]. 

### 2.4. Outcome Measures

Outcome measures selected for this review were: (1) binge eating frequency or binge remission—measured as number of binges in the last week, month or three months; (2) weight or BMI, both measured at end-of-treatment and at one-year follow-up; and (3) treatment completion rates. 

### 2.5. Meta-Analyses

As this systematic review had a special interest in the effects of BWLT as an intervention to treat BN or BED associated with overweight, we performed meta-analyses aggregating studies where BWLT was tested in one of the arms and compared with another psychological therapy, or to wait-list, or no treatment. Conducted meta-analyses examined the primary outcomes binge eating frequency (one week, one month, or three months) and weight (kg)/BMI (kg/m^2^). Additional meta-analyses investigated treatment and follow-up completion rates. 

The Review Manager Program (RevMan 5.3) was used for all analyses [25]. Mean difference analyses were conducted for continuous outcome data, together with 95% confidence intervals (CI). (Standardised mean differences were to be used where there were different measures of outcome, e.g., weight rather than BMI.) Risk ratio (RR) was used for differences in the categorical outcome of trial completion. Where more than one type of psychotherapy was included in a trial for comparison against BWLT (the treatment of main interest in this review), we have chosen the CBT arm to make comparisons as this is the therapy considered to have stronger evidence of efficacy [26]. Heterogeneity was assessed using the I-squared (I^2^) test, which provides an estimate of the percentage of variability due to heterogeneity rather than chance alone, with a value >50% considered substantial heterogeneity [24]. A random effects or fixed effects model was applied as appropriate.

Authors were contacted to provide information not available in the published study, including information needed for for quality evaluation of the trials and to obtain the results of unpublished or partly published trials. Where authors responded, it is reported with the information supplied.

## 3. Results

**Figure 1:**
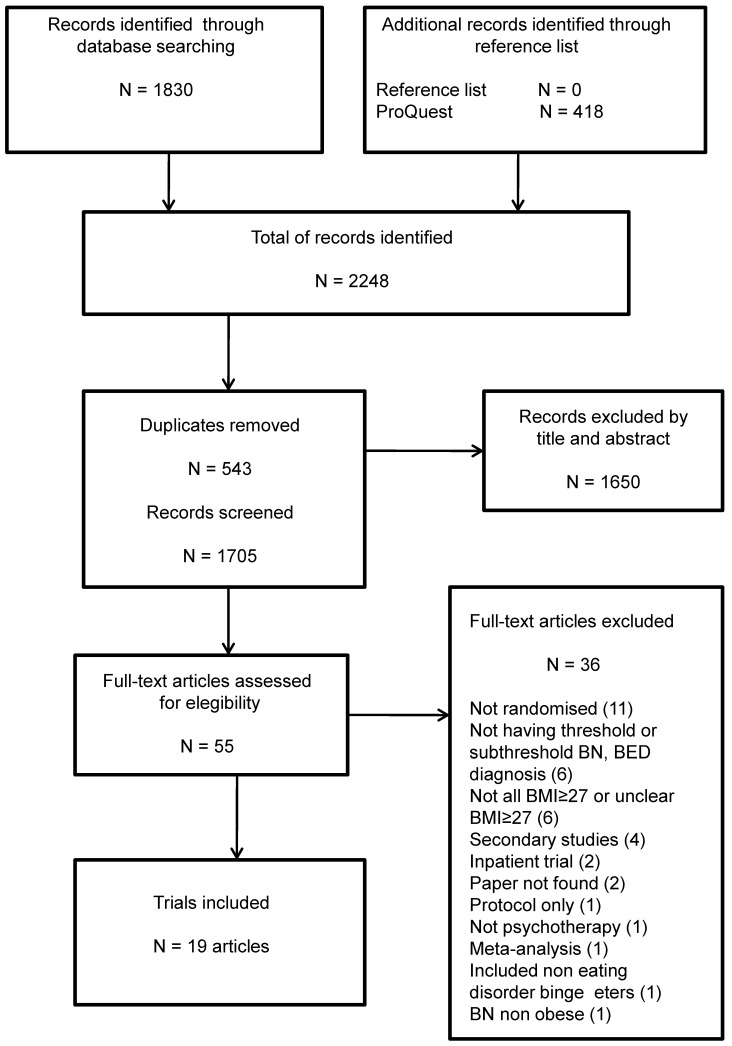
Prisma flow chart of study inclusion.

The literature search identified 2248 articles, of which 543 were duplicate papers. From 1705 papers, 1650 were excluded after title and/or abstract inspection, and 36 excluded after full article inspection. Nineteen articles were included in this review. Thirteen published articles provided full information for data extraction [17,27,28,29,30,31,32,33,34,35,36,37,38], and six further trials were included but had incomplete information, i.e. inclusion under 10% of participants with BMI ≤ 27 kg/m^2^ and/or the EDs’ diagnosis was not determined by a validated instrument [39,40,41,42,43,44]. Just one secondary study was included due to assessment of a long-term intervention (up to six-year follow-up) [35]. These results are illustrated in Figure 1. The most common reason for excluding full text articles was not being randomized (*n* = 11), followed by participants not having a diagnosis of BN, BED or subthreshold forms of these disorders (*n* = 6). A table with the excluded articles followed by the references list is added as Supplementary Material Table S1.

### 3.1. Characteristics of Included Trials

Table 1 summarizes the main features of the 19 included randomized controlled trials. All trials were reported in English and none were set outside North America or Europe [17,27,28,29,30,31,32,33,34,35,36,37,38,39,40,41,42,43,44]. Fifteen articles included participants with BED [17,27,28,29,30,33,34,35,38,39,40,41,42,43,44], and four included participants with mixed full and subthreshold BED (i.e., EDNOS or OSFED) [31,32,36,37]. There were no trials including participants with BN or subthreshold BN. The majority (*n* = 16) of the trials included both men and women, but women comprised at least 67% of participants in any trial [17,27,29,30,31,32,33,34,35,36,37,38,39,40,41,44]. Participant mean age ranged from 37.6 to 52.3 years, mean BMI ranged from 32.2 to 41.1 kg/m^2^ and two trials included a small (10% or fewer) number of participants with BMI ≤ 27 [40,44]. The sample size ranged 46–205, and all but two studies had more than 50 participants [31,41]. One trial [27] used the DSM-5 diagnostic classification system, and the remainder the DSM-IV, and none used ICD-10. The main instruments used for diagnosis were the Structured Clinical Interview for DSM-IV Axis I Disorders Patient Version (SCID-I/P) [45], and the EDE [23]. 

### 3.2. Quality Appraisal of Included Trials

Only 10 (53%) trials reported follow-up to ≥12 months. Table 2 describes the further quality appraisal of each included trial providing detailed information from studies. Seven trials reported an a priori power analysis as shown with a # symbol [17,27,28,30,32,33,36].

Table 3 summarizes the global findings regarding the methodological quality of trials included in this review, considering high, low or unclear risks of bias. The majority (*n* = 16) of trials had overall unclear risk of bias and high (*n* = 10) or unclear (*n* = 9) risk of bias due to lack of adequate blinding.

### 3.3. Results of Studies Comparing Psychological Interventions for Binge Eating Disorder Associated with Obesity

The most frequently tested intervention was CBT (15 studies) [17,28,29,30,31,32,33,34,36,37,38,39,40,41,43], followed by BWLT (*n* = 4 studies) [17,29,30,34], and IPT (2 studies) [17,38]. A form of behavioural therapy (e.g., behavioural activation and behavioural treatment) was tested in two studies [27,42]; DBT was evaluated in one trial [44]; and other types of interventions were examined in four trials [31,32,37,44].

Three trials compared CBT versus a delayed treatment and all studies reported better results for binge remission and frequency of binge eating [39,40,41] with CBT. For weight loss, two studies reported no difference between groups [40,41], and one study reported better results for weight loss favouring CBT [39]. CBT was also compared to other psychological interventions in three two-armed trials (versus CBT-spouse involvement, versus mindfulness, and versus IPT) [28,32,38], usual care in one trial [31], and CD-ROM-Based CBT in one trial [37]; in all of these studies, no between active treatment groups differences for frequency of binge eating and weight loss were reported. CBT plus low-energy-density dietary was compared to CBT plus general nutrition counselling where no differences for binge remission or weight loss were found [33]. One study investigated the effects of individual versus group CBT but found no differences in efficacy for reduction of binge eating or BMI [36]. One study compared CBT only to CBT combined with exercise, or to maintenance intervention, or to both, and reported that the groups with CBT plus exercise had greater reductions in binge remission, frequency of binge eating and BMI compared to the non-exercise groups [43]. 

In sum, in terms of end of reduction of binge eating remission or frequency, CBT was superior to a delayed treatment control in three studies, not superior to an active intervention in seven trials, and inferior to CBT combined with exercise in one trial (excluding here the BWLT trials which will be specified separately). In terms of reduction in weight/BMI, CBT was not superior to delayed treatment in two of three trials, and not superior to an active intervention in seven trials, and inferior to CBT combined with exercise in one trial [28,31,32,33,36,37,38,39,40,41,43].

Besides CBT, DBT presented better results at the end of treatment for reduction in binge frequency when compared to an active comparison group therapy (which followed a Rogerian approach) [44]. Behavioural activation compared to delayed treatment did not show differences for binge eating frequency [27]. In participants with obesity and binge eating, an intervention comparing a cognitive treatment with a behavioural treatment for obese binge eaters and obese non-binge eaters showed a higher percentage (67%) for binge abstinence with the cognitive treatment compared to the behavioural treatment in the post-treatment and six-month follow-up [42].

With regards to long-term follow-up, weight loss is being routinely reported to one year in obesity trials [18]. This review identified ten studies that reported a follow-up of at least 12 months [17,30,33,34,35,36,38,40,43,44]. Considering here only the active interventions and excluding trials with BWLT treatment (mentioned separately below), no between-group differences in frequency of binge eating, and/or binge remission rates, and/or BMI reduction were found in four trials [33,36,38,44]. The 12-month follow-up was not available in Dingemans et al. (2007) because the delayed treatment participants received the active treatment (CBT) after 24 weeks [40]. The long-term findings of the study that compared CBT associated or not with exercise and/or maintenance (four arms) found that the combined exercise groups sustained the significantly better results found at end of treatment and also at 12-month follow-up for binge eating abstinence and BMI reduction [43].

Five studies compared BWLT with active treatments and/or control group and three of these were a three-armed trial: BWLT versus CBT-GSH versus IPT [17], BWLT-GSH versus CBT-GSH versus self-monitoring control [29], and BWLT versus CBT versus CBT plus BWLT [30]. These studies reported mixed results. Two of these three-armed trials reported no significant difference between the three groups for binge eating frequency at post-treatment, but better results for binge eating remission and/or binge frequency for CBT and IPT at the follow-up period; and BWLT (including when combined with CBT) as more effective in BMI reduction at post-treatment, but not at the follow-up period [17,30]. Grilo and Masheb (2005) reported different outcomes indicating greater results for binge eating frequency in the CBT-GSH group and no differences between groups for BMI reduction [29]. However, they had no follow-up assessment. Munsch et al. (2007) conducted a trial comparing BWLT versus CBT [34], and carried out a secondary study with a six-year follow-up, where 52 remained participants from the original trial were reassessed [35]. Results of the original trial showed better results for binge remission and binge frequency in CBT group, and better results for BMI reduction in the BWLT group at end of treatment, with no difference between groups in binge remission and BMI at 12-month follow-up [34]. Finally, the Munsch’ study, with the longest follow-up (six years), reported attenuation of the results compared to post-treatment, but still an improvement with medium to large effect sizes when compared to baseline data [35]. 

Three trials with follow-up under 12 months reported no differences between the interventions in binge eating remission/frequency and BMI [28,32,37], and five trials did not have follow-up, or did not present comparative information [27,29,31,39,41].

Of note, in five trials, weight loss was reported to be significantly greater in those who achieved and sustained binge remission for a longer-term (at 6- and/or 12-month follow-up) [17,30,34,38,39].

### 3.4. Results of Meta-Analyses of Studies Comparing BWLT versus CBT

**Figure 2:**
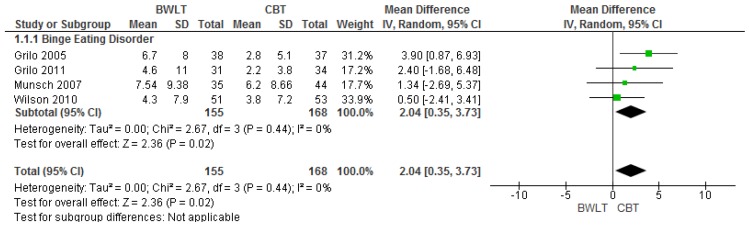
Behavioural Weight Loss Therapy versus Cognitive Behavioural Therapy-Effects on binge eating frequency at the end of treatment in individuals with binge eating disorder.

Four trials compared BWLT versus CBT [17,29,30,34] (note Munsch, 2012 was a secondary study and thus not included in this meta-analyses [35]). When aggregated in meta-analysis, a greater reduction in binge eating frequency was found with CBT when compared to BWLT at end of treatment (*n* = 4 trials, 323 participants; MD 2.04 95% CI, 0.35 to 3.73) (Figure 2).

In three further meta-analyses (Figures S1, S2 and S3), there were no significant differences for BMI at end of treatment (*n* = 4 trials, 324 participants; MD −1.01 95% CI, −3.31 to 0.29), binge eating frequency at one year follow-up (*n* = 3 trials, 260 participants; MD 1.87 95% CI, −0.08 to 3.82) or BMI at one-year follow-up (*n* = 3 trials, 261 participants; MD −0.2195% CI, −1.52 to 1.09).

Two meta-analyses (Figures S4 and S5) investigated completion rates at end of treatment (*n* = 4 trials, 375 non-completers; RR 1.16 95% CI, 0.77 to 1.74), and at one-year follow-up (*n* = 3 trials, 300 non-completers; RR 1.14 95% CI, 0.76 to 1.72) with similar attrition identified for both time-points. 

Forest plots of these latter meta-analyses are found in Supplementary Materials.

## 4. Discussion

This review aimed to examine the findings from trials where psychological therapies were tested in people with EDs co-morbid with overweight or obesity. All trials identified were of participants with BED and none included participants with BN or subthreshold BN. The most frequently tested intervention was CBT, supporting its status in guidelines as the treatment with the strongest level of evidence to support its use to treat patients with BED [26,46]. When compared with delayed treatment controls, CBT was found to be superior in reducing binge frequency but not consistently so with regards to weight loss, and not when compared to an active treatment control group. DBT was superior to an active control intervention with regards to reduced binge frequency, but this was only in one study. Cognitive therapy was superior to a behavioural intervention in reducing binge frequency in one trial.

The results of the meta-analyses comparing BWLT versus CBT showed that the latter was only favoured for reducing binge eating in the short term (end of treatment), but not at one-year follow-up. This meta-analysis concur with a review by McElroy et al. [16] for the effectiveness of CBT in reducing binge eating frequency in the short-term, but do not support their more supportive findings in the longer-term . Although BWLT showed greater effects for weight loss at the end of treatment in comparison to CBT in three trials [17,30,34], no significant differences were found between these interventions in meta-analyses, in either the short or long term. This is consistent with findings of other recent reviews [16,47].

The Pendleton trial that compared groups receiving CBT combined to instructions about exercise principles and/or self-management techniques is of particular note. This study found better results for reductions in binge days, binge abstinence and BMI in the exercising groups [43]. A systematic review also described good results for reduction in binge eating frequency and BMI can be reached when aerobic and yoga exercises are added to the treatment for BED patients [48]. Thus, greater consideration should likely be given to the addition of exercise in the psychological treatment of persons with BED associated with overweight or obesity, both in research and in clinical practice. It would also be important to report in future trials physical health outcomes beyond BMI, including engagement in and maintenance of exercise.

The consistent finding that weight loss was significantly greater in those who achieved and sustained binge remission in the long-term is also important to highlight. It indicates the need to developed approaches for weight loss treatment where reduction, and if possible abstinence of binge eating is prioritized. Further research into the relationship of these two outcomes is required to confirm this putative causal relationship.

As described in the beginning of this review, the purpose was to include trials evaluating BN associated with overweight or obesity. This review found a major gap in evidence for the management of BN comorbid with obesity, possibly due to existing controversies about potential hazards of diet restrictions in people with BN. With the rise of BN associated with overweight, there is an urgent need to develop interventions that focus on these combined conditions and demonstrate that both conditions can be safely treated.

Limitations of this systematic review are the identification of studies only in the Northern hemisphere and the underrepresentation of men in trials. In addition, several trials were found to have notable methodological limitations, including lack of ≥12- month follow-up (9 of 19 trials), unclear information about selection, performance, detection, attrition and reporting bias. The overall risk of bias was unclear for majority of trials and risk was most due to lack of blinding in trials. However, this is unlikely to change the most common result of no significant difference between active interventions, as blinding would likely reduce differences further. The more promising outcomes, albeit attenuated, in the six-year follow-up of Munsch et al. [34,35] supports the importance of longer term outcomes and suggests short term studies may be missing important differences over the longer term. 

## 5. Conclusions 

This review provides qualified support for the use of CBT in people with BED comorbid with obesity/overweight. Whilst CBT was favoured compared to BWLT for binge eating reduction in the short term, this was not the case for any other outcome and thus there was insufficient evidence to favour one approach over the other. There was also little qualitative support for CBT versus another psychological therapy in the longer term. Where CBT is not accessible, BWLT or another psychological therapy may be used. Adding exercise to CBT may enhance efficacy but more trials are needed. Higher quality trials with adequate follow-up periods are needed to test the efficacy of psychological treatments for BED and for BN with co-morbid overweight or obesity, including trials evaluating binge eating remission and weight loss in the long term.

## Figures and Tables

**Table 1:** Features of included randomized controlled trials.

Trial	Sample	Diagnostic Criteria/Classification System (Instrument)	Intervention	Time Point of Assessments Relative to the Start of the Interventions	End of Treatment Outcomes (Binge Remission/Frequency and BMI/Weight Loss)	Follow-Up of Active Treatments Outcomes (Binge Remission/Frequency and BMI/Weight Loss)
**Trials which all participants had BMI ≥ 27 and the eating disorder diagnosis was determined by a validated instrument**						
Alfonsson et al. Sweden/2015 [27]	*n* = 100# 94%♀/6%♂ Mean age 44.3 Mean BMI 41.1	BED/DSM-5 (EDE)	1. Behavioural activation 2. Delayed treatment Group sessions	Baseline EoT 10 weeks FU 3–6 months	No differences between groups in binge eating frequency. No BMI results.	Only for active treatment – no comparison.
Gorin et al. USA/2003 [28]	*n* = 94# 100%♀ Mean age 45.2 Mean BMI 39.42	BED/DSM-IV (SCID-I/P)	1. Standard cognitive behavioural therapy 2. CBT-spouse involvement 3. Delayed treatment Group sessions	Baseline EoT 12 weeks FU 6 months	Comparison between active treatments: no differences in binge eating frequency or BMI. Active treatments compared to wait-list: better results for binge eating frequency and BMI for CBT groups.	Only comparison between active treatments: no differences in binge eating frequency or BMI.
Grilo and Masheb USA/2005 [29]	*n* = 90 79%♀/21♂ Mean age 46.3 Mean BMI 35.5	BED/DSM-IV (SCID-I/P+EDE)	1. CBTgsh 2. BWLgsh 3. Self-monitoring control Individual sessions	Baseline EoT 12 weeks	CBTgsh had greater results for binge eating frequency compared to BWLgsh and the control. No differences between groups for BMI.	No follow-up.
Grilo et al. USA/2011 [30]	*n* = 125# 67%♀/33%♂ Mean age 44.8 Mean BMI 38.8	BED/DSM-IV (SCIDI/P+ EDE)	1. CBT 2. BWL 3. CBT + BWL Group sessions	Baseline EoT 24 weeks (CBT and BWL) FU 6–12 months	No differences between groups in binge remission and binge eating frequency. BWLT and CBT + BWL had significant greater per cent BMI loss than CBT.	No differences between groups in binge remission rates. Binge eating frequency significantly lower in CBT than BWL at both follow-up. At six-month significant BMI loss in BWL than CBT, but not sustained at 12-month.
Grilo et al. USA/2013 [31]	*n* = 48 79%♀/21%♂ Mean age 45.8 Mean BMI 37.62	Threshold and subthreshold BED/DSM-IV (SCID-I/P + EDE)	1. Self-help CBT 2. Usual care Self-help	Baseline EoT 4 months	No differences between groups in binge remission, frequency of binge eating or BMI.	No follow-up.
Kristeller et al. USA/2014 [32]	*n* = 150# 88%♀/12%♂ Mean age 46.5 Mean BMI 40.2	Threshold and subthreshold BED/DSM-IV-R (EDE)	1. Mindfulness-based eating awareness training 2. Psychoeducational cognitive-behavioural treatment 3. Delayed treatment Group sessions	Baseline EoT 1 month FU 4 months	Comparison between active treatments: no differences in binge eating frequency or BMI. Active treatments compared to wait-list: better results for binge eating frequency and BMI for both active interventions.	Same results found at end of treatment.
Masheb et al. USA/2011 [33]	*n* = 50# 76%♀/24♂ Mean age 45.8 Mean BMI 39.1	BED/DSM-IV-TR (SCID-I/P+EDE)	1. CBT + low-energy- density diet 2. CBT + general nutrition Individual sessions	Baseline EoT 6 months FU 12 months	No differences between groups in binge remission or BMI.	Same results found at end of treatment.
Munsch et al. Switzerland/2007 [34] and Munsch et al. Switzerland/2012 [35]	*n* = 80 89%♀/11%♂ Mean age 46.1 Mean BMI 34.0 *n* = 52 90%♀/10%♂ Mean age 52.3 Mean BMI 32.5	BED/DSM-IV-TR (EDE) BED/DSM-IV-TR (EDE-Q)	1. CBT 2. BWLT Group sessions	Baseline EoT 4 months FU 12 months FU 6 years	CBT improved significantly for binge remission and binge eating frequency. However, BWLT was significantly better in weight loss.	No differences between groups in binge remission, binge eating frequency or BMI. Comparing the end of treatment to six-year follow-up, these outcomes significantly worsened. Comparing the baseline to six-year follow-up these measures still improved with medium to large effect sizes.
Ricca et al. Italy/2010 [36]	*n* = 144# 86%♀/14%♂ Mean age 46.9 Mean BMI 38.1	Threshold and subthreshold BED/DSM-IV (SCID-I/P)	1. Individual CBT 2. Group CBT	Baseline EoT 24 weeks for I-CBT 22 weeks for G-CBT FU 3 years	No differences between groups in reduction of binge eating episodes and BMI.	Same results found at end of treatment.
Shapiro et al. USA/2007 [37]	*n* = 66 92%♀/8%♂ Mean age 39.5 Mean BMI 37.3	Threshold and subthreshold BED/DSM-IV (SCID-I/P)	1. Group CBT 2. CD-ROM 3. Delayed treatment	Baseline EoT 10 weeks FU 2 months	No differences between groups in binge eating frequency or BMI.	Same results found at end of treatment.
Wilfley et al. USA/2002 [38]	*n* = 162 83%♀/17%♂ Mean age 45.2 Mean BMI 37.4	BED/DSM-IV (SCID for DSM-III-R + EDE)	1. CBT 2. Interpersonal therapy Group sessions + three individual sessions	Baseline EoT 20 weeks FU 4–8–12 months	No differences between groups in binge eating frequency or BMI.	Same results found at end of treatment.
Wilson et al. USA/2010 [17]	*n* = 205# 79%♀/21%♂ Mean age 48.4 Mean BMI 36.4	BED/DSM-IV (SCID-I + EDE)	1. CBTgsh 2. Interpersonal therapy 3. BWLT Individual sessions for Interpersonal therapy and BWLT	Baseline EoT 24 weeks FU 12–24 months	No differences between groups in binge remission and binge eating frequency. BWLT was significantly more effective in BMI reduction than the two other treatments.	1-year FU: no differences between groups in measures of binge eating and more significant BMI gain for the BWL group compared to CBTgsh group. Two-year FU: IPT and CBTgsh were more effective for remission of binge episodes. No difference for BMI comparing all groups.
**Trials which included under 10% participants with BMI ≤ 27 and/or the eating disorder diagnosis was not determined by a validated instrument**						
Agras et al. USA/1995 [39]	*n* = 50 86%♀/14%♂ Mean age 47.6 Mean BMI 37.1	BED/not specified (Structured clinical interview)	1. CBT 12 weeks followed by 12 weeks of either IPT for non -responders or weight loss therapy for responders to CBT 2. Delayed treatment Group sessions	Baseline EoT 24 weeks	Active treatment compared to wait-list: better results for binge eating frequency and BMI for active intervention. IPT group: binge eating increased and weight increased with IPT not significant. Weight loss therapy: significant weight loss and maintained reduced binge eating.	No follow-up.
Dingemans et al. Netherlands/ 2007 [40]	*n* = 52 94%♀/6%♂ Mean age 37.6 Mean BMI 39.0 *n* = BMI < 27 *	BED/DSM-IV (Instrument not specified)	1. CBT 2. Delayed treatment Group sessions	Baseline EoT 20 weeks FU 12 months	Significant binge remission and reduction in frequency in binge eating in CBT group. No significant BMI change.	Only for active treatment—no comparison.
Eldredge et al. USA/1997 [41]	*n* = 46 96%♀/4%♂ Mean age 45.2 Mean BMI 38.4	BED (No other information)	1. CBT 2. Delayed treatment Group sessions	Baseline EoT 12 weeks FU 24 weeks	Significant binge remission and reduction in frequency in binge eating in CBT group. No significant BMI change.	No information.
Nauta et al. Netherlands/2000 [42]	*n* = 74 100% ♀ Mean age 38.3 Mean BMI 33.1 Mixed sample of binge eating and non-binge eating participants	BED/DSM-IV (Structured interview)	1. Cognitive treatment 2. Behavioural treatment Group sessions	Baseline EoT 15 weeks FU 6 months	At post-treatment 67% binge abstinence with cognitive treatment vs. 44% abstinence with behavioural treatment in obese binge eating participants. Other outcomes not reported separately for binge eating participants.	86% binge abstinence with cognitive treatment vs. 44% abstinence with behavioural treatment in obese binge eating participants (significant *p* < 0.01).
Pendleton et al. USA/2002 [43]	*n* = 114 100% ♀ Mean age 45.0 Men BMI 36.2	BED (no other information)	1. CBT + exercise + maintenance 2. CBT + exercise − maintenance 3. CBT − exercise + maintenance 4. CBT − exercise − maintenance Group sessions	Baseline EoT 4 months FU 6–12 months	Exercisers had significantly greater reduction in binge frequency and BMI compared to non-exercisers groups. Addition of the maintenance program did not influence on binge eating behaviour but influenced changes in BMI.	Same results found at end of treatment.
Safer et al. USA/2010 [44]	*n* = 101 85% ♀/15%♂ Mean age 52.2 Mean BMI 36.3 *n* = 9 BMI < 27 *	BED/DSM-IV (EDE)	1. Dialectical behaviour therapy adapted for binge eating (DBT-BED) 2. Active comparison group therapy (ACGT) Group sessions	Baseline EoT 21 weeks FU 3, 6, and 12 months	DBT-BED group achieved significant reduction in binge frequency than ACGT group. No differences found between groups for BMI.	No differences between groups in binge eating frequency or BMI.

Note: BED: binge eating disorder; BMI: body mass index; BWLgsh: behavioural weight loss – guided self-help; BWLT: behavioural weight loss treatment; CBT: cognitive behavioural therapy; CBTgsh: cognitive-behavioural therapy—guided self-help; EDE: eating disorder examination; EDE-Q: eating disorder examination questionnaire; DSM: diagnostic and statistical manual of mental disorders; EoT: end of treatment; FU: follow-up; SCID-I/P: Structured Clinical Interview for DSM-IV Axis Disorders Patient Version; * Unpublished information on participant BMI numbers provided by author; #: Included a priori power analysis; ♀: women, ♂: men.

**Table 2:** Quality appraisal of the nineteen included trials.

Reference	Randomisation and Allocation Concealment	Blinding	Treatment Attrition	Reporting Bias	Overall Risk of Bias (Modal Assessment)
**Trials which all participants had BMI > 27 kg/m^2^ and the eating disorder diagnosis was determined by a validated instrument**					
Alfonsson et al. # 2015 [27]	Adequate randomisation Unclear allocation concealment Unclear RoB	No blinding High RoB	EoT 32% Unclear RoB	Used ITT MEM Trial registration not reported Unclear RoB	Unclear
Gorin et al. # 2003 [28]	Unclear randomisation and allocation concealment Unclear RoB	No blinding High RoB	EoT not reported Assessment attrition 34% Unclear RoB	Used ITT/LOCF Participant flow not reported Trial registration not reported Unclear RoB	Unclear
Grilo and Masheb 2005 [29]	Adequate randomisation and allocation concealment Low RoB	No blinding except for participant expectations High RoB	EoT 22% Attrition higher (33%) in BWLgsh Unclear RoB	Used ITT/LOCF Participant flow reported Trial registration not reported Unclear RoB	Unclear
Grilo et al. # 2011 [30]	Adequate randomisation Unclear allocation concealment Unclear RoB	No blinding High RoB	EoT 31% Unclear RoB	Used ITT/MEM Participant flow reported Trial registered, outcomes unchanged from protocol Low RoB	Unclear
Grilo et al. 2013 [31]	Adequate randomisation and allocation concealment Low Rob	Outcome assessment assessor blind Participants could not be blind Unclear RoB	No EoT attrition Low RoB	Used ITT/MEM Participant flow reported Trial registration not reported Unclear RoB	Unclear
Kristeller et al. # 2014 [32]	Inadequate randomisation Unclear allocation concealment High RoB	Unclear blinding Unclear RoB	Attrition 30% greater in control conditions but not significant Unclear RoB	Used ITT/MEM Participant flow reported Trial registration not reported Unclear RoB	Unclear
Masheb et al.# 2011 [33]	Adequate randomisation and allocation concealment Low Rob	Outcome assessment assessor blind Participants not reported blind Unclear RoB	EoT 14% Low RoB	Used ITT/MEM/LOCF Participant flow reported Trial registration reported Low RoB	Low
Munsch et al. 2007 [34] /2012 [35]	Unclear randomisation and allocation concealment Unclear RoB	Outcome assessment assessor not blind Participants blind unclear High RoB	EoT 27.5% Unclear RoB	Used ITT/LOCF and completer for univariate models Participant flow reported Trial registration not reported Unclear RoB	Unclear
Ricca et al. # 2010 [36]	Adequate randomisation and allocation concealment Low Rob	Outcome assessment assessor blind Participants could not be blind Unclear RoB	EoT 4.9% Low RoB	Used ITT/LOCF Participant flow reported Trial registration not reported Unclear RoB	Unclear
Shapiro et al. 2007 [37]	Unclear randomisation and allocation concealment Unclear RoB	Outcome assessment not blind Participants could not be blind. High RoB	EoT 41% High RoB	Used ITT/MEM Participant flow not reported Trial registration not reported Unclear RoB	High
Wilfley et al. 2002 [38]	Unclear randomisation and allocation concealment Unclear RoB	Blinding only for treatment fidelity assessment Assessors not consistently blind Participants blind unclear High RoB	EoT 9.9% Low RoB	Used ITT method unclear and completer analyses Participant flow reported Retrospective trial registered Unclear RoB	Unclear
Wilson et al. # 2010 [17]	Adequate randomisation Unclear allocation concealment Unclear RoB	Outcome assessment assessor blind Participants blinding unclear Unclear RoB	EoT 23.1% EoT Attrition 7% IPT/28% BWL/30% CBTgsh IPT significant lower attrition Unclear RoB	Used ITT/MEM Participant flow reported Trial registration reported Low RoB	Unclear
**Trials which either did not use a validated instrument for eating disorder diagnosis and/or included under 10% participants with BMI < 27 kg/m^2^**					
Agras et al. 1995 [39]	Unclear randomisation and allocation concealment Unclear RoB	Unclear blinding Unclear RoB	EoT 12.8% Low RoB	Used ITT/LOCF and completer only analysis Participant flow not reported Trial registration not reported Unclear RoB	Unclear
Dingemans et al. 2007 [40]	Unclear randomisation Adequate allocation concealment Unclear RoB	Assessor blind to group Participants could not be blind Unclear RoB	EoT 7% Low RoB	Used MLA analysis Participant flow reported Trial registration not reported Unclear RoB	Unclear
Eldredge et al. 1997 [41]	Unclear randomisation and allocation concealment Unclear RoB	No blinding High RoB	EoT 19% Low RoB	No ITT reported Participant flow not reported Trial registration not reported High RoB	High
Nauta et al. 2000 [42]	Unclear randomisation and allocation concealment Unclear RoB	Blind not reported High RoB	EoT 13.5% Low RoB	Used ITT repeated-measures multivariate and univariate analysis of variance Participant flow not reported Trial registration not reported Unclear RoB	Unclear
Pendleton et al. 2002 [43]	Unclear randomisation and allocation concealment Unclear RoB	Unclear blinding Unclear RoB	EoT attrition unclear Assessment attrition 22.8% Unclear RoB	Not used ITT Participant flow not reported Trial registration not reported High RoB	Unclear
Safer et al. 2010 [44]	Unclear randomisation and allocation concealment Unclear RoB	No blinding reported Unclear RoB	EoT 19% but higher 33% in control group and 10% in DBT Unclear RoB	Used linear mixed models analysis Participant flow reported Trial registered Low RoB	Unclear

Note: DBT: dialectical behaviour therapy; EoT: end of treatment; GEE: generalized estimating equation; BWLgsh: guided self-help behavioural weight loss; ITT: intention-to-treat; LOCF: last observation carried forward; MEM: mixed effect model; MLA: multilevel analysis; #: Included a priori power analysis.

**Table 3:** A synthesis of the quality appraisal of the nineteen included trials.

	High Risk	Low Risk	Unclear Risk
**Randomisation and Allocation Concealment**	*n* = 1	*n* = 4	*n* = 14
	[32]	[29,31,33,36]	[17,27,28,30,34,35,37,44]
**Blinding**	*n* = 10	*n* = 0	*n* = 9
	[27,28,29,30,34,35,37,38,41,42]		[17,31,32,33,36,39,40,43,44]
**Treatment Attrition**	*n* = 1	*n* = 8	*n* = 10
	[37]	[31,33,36,38,39,40,41,42]	[17,27,28,29,30,32,34,35,43,44]
**Reporting Bias**	*n* = 2	*n* = 4	*n* = 13
	[41,43]	[17,30,33,44]	[27,28,29,31,32,34,35,36,37,38,39,40,42]
**Overall Risk of Bias (Modal Assessment)**	*n* = 2	*n* = 1	*n* = 16
	[37,41]	[33]	[17,27,28,29,30,31,32,34,35,36,38,39,40,42,43,44]

## Supplementary Materials

The following are available online at http://www.mdpi.com/2072-6643/9/3/299/s1. Table S1: Trials excluded from the review with reasons for exclusion, Figures S1: Forest plot of additional meta-analysis: Mean BAO at end of treatment, Figure S2: Forest plot of additional meta-analysis: Mean binge frequency at 12 months follow-up, Figure S3: Forest plot of additional meta-analysis: Mean BMI at 12 months follow-up, Figure S4: Forest plot of additional meta-analysis: Treatment completion rates at end treatment, Figure S5: Forest plot of additional meta-analysis: Treatment completion rates at end treatment.

## Abbreviations

BN	Bulimia Nervosa
BED	Binge Eating Disorder
BMI	Body Mass Index
BWLT	Behavioural Weight Loss Therapy
CBT	Cognitive Behavioural Therapy
CBT-E	Cognitive Behavioural Therapy- Enhanced
CD-ROM	Compact Disc Read-Only Memory
CI	Confidence Intervals
DBT	Dialectical Behaviour Therapy
DSM-IV-TR	Diagnostic and Statistical Manual of Mental Disorders, 4th Edition, Text Revision
DSM-5	Diagnostic and Statistical Manual of Mental Disorders, 5th Edition
EDs	Eating Disorders
EDE	Eating Disorder Examination
EDNOS	Eating Disorder Not Otherwise Specified
EMBASE	Excerpta Medica dataBASE
ICD-10	International Classification of Diseases, 10th revision
ICD-11	International Classification of Diseases, 11th revision
LILACS	Literatura Latino-Americana e do Caribe em Ciências da Saúde
MEDLINE	Medical Literature Analysis and Retrieval System Online
NCDs	Non communicable diseases
OSFED	Other Specified Feeding or Eating Disorder
PRISMA	Preferred Reporting Items for Systematic Reviews and Meta-Analyses
RCT	Randomised Controlled Trial
REVMAN	Review Manager
SCID-I/P	Structured Clinical Interview for DSM-IV-TR Axis I Disorders, Research Version, Patient Edition
SMD	Standardized Mean Difference
WHO	World Health Organization
