# Supplementary material for: The Efficacy of Psychological Therapies in Reducing Weight and Binge Eating in People with Bulimia Nervosa and Binge Eating Disorder Who Are Overweight or Obese—A Critical Synthesis and Meta-Analyses
Source: Nutrients. 2017 Mar 17;9(3):299. doi: 10.3390/nu9030299 (PMC5372962; doi:10.3390/nu9030299)

Table S1: Trials excluded from the review with reasons for exclusion.

| Number | Reference | Reason for exclusion |
| --- | --- | --- |
| 01 | Annunziato et al.  2009 | No ED diagnosis/ Wrong diagnosis |
| 02 | Blaine & Rodman  2007 | Meta-analysis/ Not an intervention |
| 03 | Burton & Stice  2006 | Not all BMI≥27 |
| 04 | Carrard et al.  2011 | Not all BMI ≥27 |
| 05 | Castelnuovo et al.  2011 | Inpatient Trial |
| 06 | Cesa et al.  2013 | Inpatient Trial |
| 07 | DeBar et al.  2011 | Not a study of individuals withBN/BED |
| 08 | Delinsky et al.  2006 | No ED diagnosis/ Wrong diagnosis |
| 09 | De Zwaan et al.  2012 | Protocol only |
| 10 | Downe et al.  2009 | Not PsychotherapyTrial/ Wrong intervention |
| 11 | Fairburn et al.  1995 | BN non obese |
| 12 | Fischer et al.  2014 | Included 15.4% BMI < 27 Author replied |
| 13 | Fossati et al.  2004 | Not randomised |
| 14 | Grilo et al.  2012 | Not randomised |
| 15 | Grilo et al.  2013 | Not randomised |
| 16 | Hilbert et al.  2007 | Secondary study |
| 17 | Hoie et al.  1997 | Not randomised |
| 18 | Kalman et al.  2002 | Not randomised |
| 19 | Marchesini et al.  2002 | Not randomised |
| 20 | Masheb et al.  2007 | Secondary study |
| 21 | Masheb & Grilo  2008 | Secondary study |
| 22 | Melchionda et al.  2003 | No ED diagnosis/ Wrong diagnosis |
| 23 | Minniti et al.  2007 | No ED diagnosis/ Wrong diagnosis |
| 24 | Peterson et al.  2009 | Unclear BMI≥ 27/ Author did not reply |
| 25 | Porzelius et al.  1995 | No ED diagnosis |
| 26 | Prince et al.  2013 | Paper not found |
| 27 | Ramanouskaya  2014 | Paper not found |
| 28 | Riva et al.  2000^a^ | Not randomised |
| 29 | Riva et al.  2000^b^ | Not randomised |
| 30 | Riva et al.  2001 | Not randomised |
| 31 | Riva et al.  2002 | Not randomised |
| 32 | Robinson et al.  2012 | Secondary study |
| 33 | Schlup et al.  2009 | Unclear BMI≥ 27/ Author did not reply |
| 34 | Shelley & Mac Millan  2007 | No ED diagnosis/ Wrong diagnosis |
| 35 | Thackwray et al.  1993 | Unclear BMI≥ 27 |
| 36 | Traverso et al.  2000 | Not randomised |

**References of Excluded Trials**

01. Annunziato, R.A.; Timko, C.A.; Crerand, C.E.; Didie, E.R.; Bellace, D.L.; Phelan, S.;

Kerzhnerman, I.; Lowe, M.R. A randomized trial examining differential meal

replacement adherence in a weight loss maintenance program after one-year

follow-up. *Eat Behav* **2009**, 10, 176-183.

02. Blaine, B.; Rodman, J. Responses to weight loss treatment among obese

individuals with and without BED: a matched-study meta-analysis. *Eat Weight*

*Disord* **2007**, 12, 54-60.

03. Burton, E.; Stice, E. Evaluation of a healthy-weight treatment program for bulimia

nervosa: a preliminary randomized trial. *Behav Res Ther* **2006**, 44, 1727-1738.

04. Carrard, I.; Crépin, C. Rouget, P. Lam, T.; Golay, A. Van der Linden, M.

Randomised controlled trial of a guided self-help treatment on the Internet for binge

eating disorder. *Behav Res Ther* **2011**, 49, 482-491.

05. Castelnuovo, G.; Manzoni, G.M.; Villa, V.; Cesa, G.L.; Molinari, E. Brief strategic

therapy vs cognitive behavioral therapy for the inpatient and telephone-based

outpatient treatment of binge eating disorder: the STRATOB randomized controlled

clinical trial. *Clin Pract Epidemiol Ment Health* **2011**, 7,29-37.

06. Cesa, G.L.; Manzoni, G.M.; Bacchetta, M.; Castelnuovo, G.; Conti, S.; Gaggioli, A.;

Mantovani, F.; Molinari, E.; Cárdenas-López, G.; Riva, G. Virtual reality for

enhancing the cognitive behavioral treatment of obesity with binge eating disorder:

randomized controlled study with one-year follow-up. *J Med Internet Res* **2013**,

12:e113, DOI 10.2196/jmir.2441.

07. DeBar, L.L.; Striegel-Moore, R.H.; Wilson, G.T.; Perrin, N.; Yarborough, B.J.

Dickerson, J.; Lynch, F.; Rosselli, F.; Kraemer, H.C. Guided self-help treatment for

recurrent binge eating: replication and extension. *Psychiatr Serv* **2011**, 62, 367-

373.

08. Delinsky, S.S.; Latner, J.D.; Wilson, G.T. Binge eating and weight loss in a self-help

behavior modification program. *Obesity (Silver Spring)* **2006**, 14, 1244-1249.

09. de Zwaan, M.; Herpertz, S.; Zipfel, S.; Tuschen-Caffier, B.; Friederich, H.C.;

Schmidt, F.; Gefeller, O.; Mayr, A.; Lam, T.; Schade-Brittinger, C.; Hilbert, A.

INTERBED: internet-based guided self-help for overweight and obese patients with

full or subsyndromal binge eating disorder. A multicenter randomized controlled

trial. *Trials* **2012**, 21, 220, DOI 10.1186/1745-6215-13-220.

10. Downe, K.A.; Goldfein, J.A.; Devlin, M.J. Restraint, hunger, and disinhibition

following treatment for binge-eating disorder. *Int J Eat Disord* **2009**, 42, 498-504.

11. Fairburn, C.G.; Norman, P.A.; Welch, S.L.; O´Connor, M.E.; Doll, H.A. Peveler,

R.C. A prospective study of outcome in bulimia nervosa and the long-term effects

of three psychological treatments. *Archives of General Psychiatry* **1995**, 52, 304-

312.

12. Fischer, S.; Meyer, A.H.; Dremmel, D.; Schlup, B.; Munsch, S. Short-term

cognitive-behavioral therapy for binge eating disorder: long-term efficacy and

predictors of long-term treatment success. *Behav Res Ther* **2014**, 58, 36-42.

13. Fossati, M.; Amati, F.; Painot, D.; Reiner, M.; Haenni, C.; Golay, A. Cognitive-

behavioral therapy with simultaneous nutritional and physical activity education in

obese patients with binge eating disorder. *Eat Weight Disord* **2004**, 9, 134-138.

14. Grilo, C.M.; White, M.A.; Wilson, GT.; Gueorguieva, R.; Masheb, R.M. Rapid

response predicts 12-month post-treatment outcomes in binge-eating disorder:

theoretical and clinical implications. *Psychol Med* **2012**, 42, 807-817.

15. Grilo, C.M.; White, M.A.; Gueorguieva, R.; Wilson, GT.; Masheb, R.M. Predictive

significance of the overvaluation of shape/weight in obese patients with binge

eating disorder: findings from a randomized controlled trial with 12-month follow-up.

*Psychol Med* **2013**, 43, 1335-1344.

16. Hilbert, A.; Saelens, B.E.; Stein, R.I.; Mockus, D.S.; Welch, R.R.; Matt, G.E.;

Wilfley, D.E. Pretreatment and process predictors of outcome in interpersonal and

cognitive behavioral psychotherapy for binge eating disorder. *J Consult Clin*

*Psychol* **2007**, 75, 645-651.

17. Hoie, L.H.; Myking, E.; Reine, E.C.; Bruusgaard, D. Diet and exercise in addition to

psychotherapy, in the treatment of patients suffering from eating disorders with

obesity. *Eat Weight Disord* **1997**, 2, 207-210.

18. Kalman, D.; Cascarano, H.; Krieger, D.R.; Incledon, T.; Woolsey, M. Frequency of

binge eating disorder in an outpatient weight loss clinic. *J Am Diet Assoc* **2002**,

102, 697-699.

19. Marchesini, G.; Natale, S.; Chierici, S.; Manini, R.; Besteghi, L.; Di Domizio, S.;

Sartini, A.; Pasqui, F.; Baraldi, L.; Forlani, G.; Melchionda, N. Effects of cognitive-

behavioural therapy on health-related quality of life in obese subjects with and

without binge eating disorder. *Int J Obes Realt Metab Disord* **2002**, 26, 1261-1267.

20. Masheb, R.M.; Grilo, C.M. Rapid response predicts treatment outcomes in binge

eating disorder: implications for stepped care. *J Consult Clin Psychol* **2007**, 75,

639-644.

21. Masheb, R.M.; Grilo, C.M. Prognostic significance of two sub-categorization

methods for the treatment of binge eating disorder: negative affect and

overvaluation predict, but do not moderate, specific outcomes. *Behav Res Ther*

**2008**, 46, 428-437.

22. Melchionda, N.; Besteghi, L.; Di Domizio S.; Pasqui F.; Nuccitelli, C.; Migliorini, S.;

Baraldi, L.; Natale, S.; Manini, R.; Bellini, M.; Belsito, C.; Forlani, G.; Marchesini, G.

Cognitive behavioural therapy for obesity: one-year follow-up in a clinical setting.

*Eat Weight Disord* **2003**, 8, 188-193.

23. Minniti, A.; Bissoli, L.; Di Francesco, V.; Fantin, F.; Mandragona, R.; Olivieri, M.;

Fontana, G.; Rinaldi, C.; Bosello, O.; Zamboni, M. Individual versus group therapy

for obesity: comparison of dropout rate and treatment outcome. *Eat Weight Disord*

**2007**, 12, 161-167.

24. Peterson, C.B.; Mitchell, J.E.; Crow, S.J.; Crosby, R.D.; Wonderlich, S.A. The

efficacy of self-help group treatment and therapist-led group treatment for binge

eating disorder. *Am J Psychiatry* **2009**, 166, 1347-1354.

25. Porzelius, L.K.; Houston, C.; Smith, M.; Arfken, C.; Fisher Jr., E. Comparison of a

standard behavioral weight loss treatment and a binge eating weight loss

treatment. *Behav Ther* **1995**, 26, 119-134.

26. Prince, M.; Young, D.; Mazoyer, M.; Gilchrist, L.; Forde, L. Developing a new

treatment approach to binge eating and weight management. *Clinical Psychology*

*Forum*  **2013**, 244, 49-52.

<http://www.nhsggc.org.uk/media/231776/BPS%20Annual%20Conf%202013.pdf>

(accessed on 21 March 2016).

27. Ramanouskaya. Comparison of individual and group cognitive behavioral therapy

for binge eating disorder. A randomized, two-year follow-up study (Poster). 21th

European Congress on Obesity (ECO2014), Sofia, Bulgaria, May 28, 2014.

<https://www.karger.com/Article/Abstract/363668> (accessed on 21 March 2016).

28. Riva, G.; Bacchetta, M.; Baruffi, M.; Cirillo, G.; Molinari, E. Virtual reality

environment for body image modification: a multidimensional therapy for the

treatment of body image in obesity and related pathologies. *Cyber Psychology &*

*Behavior* **2000**, 3, 421-431.

29. Riva, G.; Bacchetta, M.; Baruffi, M.; Rinaldi, S.; Vincelli, F.; Molinari, E. Virtual

reality-based experiential cognitive treatment of obesity and binge-eating disorders.

*Clin Psychol Psychot* **2000**, 7, 209-219.

30. Riva, G.; Bacchetta, M.; Baruffi, M.; Molinari, E. Virtual reality-based

multidimensional therapy for the treatment of body image disturbances in obesity:

a controlled study. *Cyber Psychology & Behavior* **2001**, 4, 511-526.

31. Riva, G.; Bacchetta, M.; Baruffi, M.; Molinari, E. Virtual reality-based

multidimensional therapy for the treatment of body image disturbances in binge

eating disorders: a preliminary controlled study. *IEEE*  **2002**, 6, 224-234.

32. Robinson, A.H.; Safer, D.L. Moderators of dialectical behavior therapy for binge

eating disorder: results from a randomized controlled trial. *Int J Eat Disord***2012**,

45, 597-602.

33. Schlup, B.; Munsch, S.; Meyer, A.H.; Margraf, J.; Wilhelm, F.H. The efficacy of a

short version of a cognitive-behavioral treatment followed by booster sessions for

binge eating disorder. *Behav Res Ther* **2009**, 47, 628-635.

34. Shelley-Ummenhofer, J.; MacMillan, P.D. Cognitive-behavioural treatment for

women who binge eat. *Can J Diet Pract Res* **2007**, 68, 139-142.

35. Thackwray, D.E.; Smith, M.C.; Bodfish, J.W.; Meyers, A.W. A comparison of

behavioral and cognitive-behavioral interventions for bulimia nervosa. *J Consult*

*Clin Psychol* **1993**, 61, 639-645.

36. Traverso, A.; Ravera, G.; Lagattolla, V.; Testa, S.; Adami, G.F. Weight loss after

dieting with behavioral modification for obesity: the predicting efficiency of some

psychometric data. *Eat Weight Disord* **2000**, 5, 102-107.

**Figure S1.Forest plot of additional meta-analysis: mean BMI at end of treatment**

Meta-analysis 2 Mean BMI at end of treatment


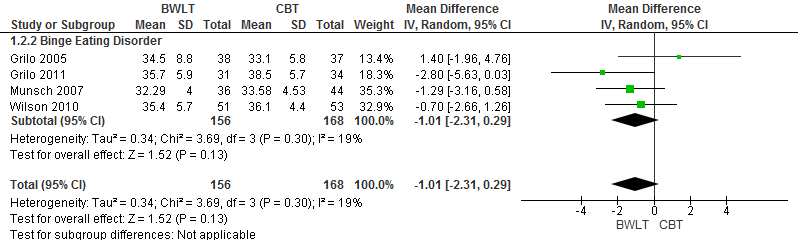


**Figure S2. Forest plot of additional meta-analysis: Mean binge frequency at 12 months follow-up**


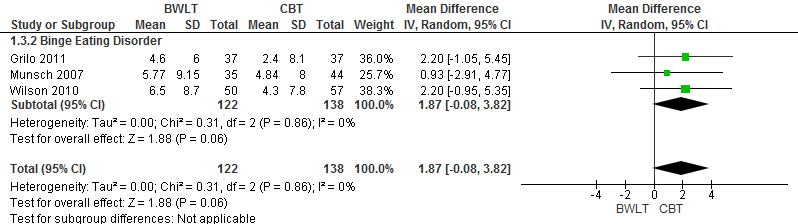


**Figure S3. Forest plot of additional meta-analysis: mean BMI at 12 months follow-up**


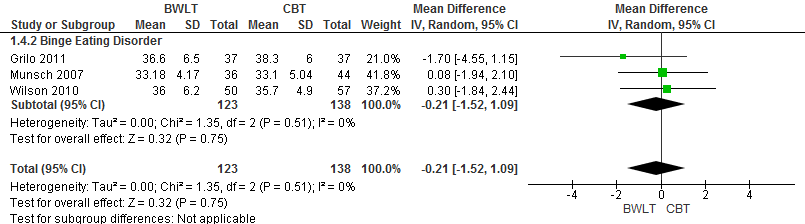


**Figure S4. Forest plot of additional meta-analysis: Treatment completion rates at end treatment**


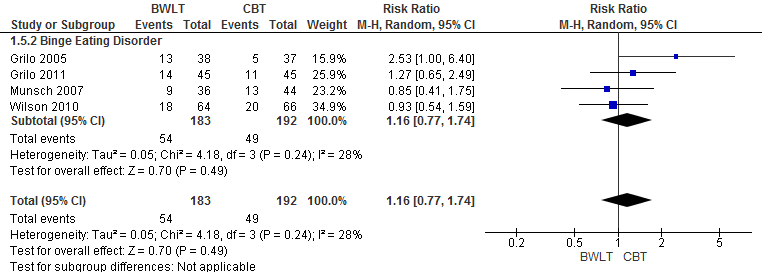


**Figure S5. Forest plot of additional meta-analysis: Treatment completion rates at end treatment**


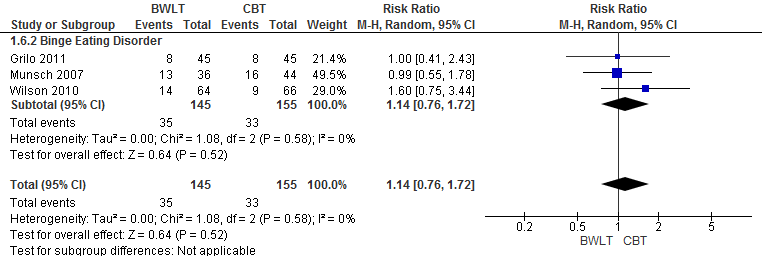

Supplement: Supplementary file 1 [file nutrients-09-00299-s001.docx]
